# Home range size and habitat use of the blue-crowned laughingthrush during the breeding season

**DOI:** 10.7717/peerj.8785

**Published:** 2020-04-21

**Authors:** Tao Liu, Yongtao Xu, Bai Mo, Jinze Shi, Yachang Cheng, Weiwei Zhang, Fumin Lei

**Affiliations:** 1 College of Forestry, Jiangxi Agricultural University, Nanchang, Jiangxi, China; 2 Department of Migration, Max Planck Institute of Animal Behavior, Radolfzell, Germany; 3 University of Konstanz, Department of Biology, Konstanz, Germany; 4 Institute of Zoology, Chinese Academy of Sciences, Beijing, China

**Keywords:** Garrulax courtoisi, Radio telemetry, Home range, Habitat use

## Abstract

The home range size and habitat use of the blue-crowned laughingthrush (*Garrulax courtoisi*, hereafter BCLT), a critically endangered, subtropical, cooperative-breeding bird species in southeast China, were studied during its breeding period using radio telemetry at different sites during 3 consecutive years (2016–18, from May to June of each year). A total of 17 birds (12 males, four females, and one of unknown sex) were tagged, and a total 1515 locations (mean ± se = 89.12 ± 11.42) were obtained over 54 days of tracking. The average 100% minimum convex polygon (MCP) home range size was 10.05 ± 1.17 ha, and the estimated KDE core area (fiexed kernel density estimator, KDE) size was 7.84 ± 1.18 ha. According to the Wilcoxon rank sum tests, both the 100% MCP and KDE core area size of males did not significantly differ from those of females. There were no significant differences in the 100% MCP or KDE core area sizes of the three breeding sites. The available habitats in the breeding sites included water areas, shrubs, grass plots, woodland, residential areas, vegetable field, farmland, and sandy beaches; among them, only woodland was significantly preferred by BCLTs. Woodland (average use ratio was 45.86 ± 1.74%) was strongly preferred by BCLTs for nesting, foraging and roosting. Shrubs/grass plots (24.72 ± 3.39%) and vegetable plots (11.80 ± 1.83%) were used relatively more often than the other habitats, except woodland, since shrubs were always used as perches, and vegetable plots were rich in food resources. Vertically, the canopy layer was used most often from April to June, but it was used most in May when the birds were hatching and brooding. This result indicates that BCLT is predominantly active in the upper strata during the breeding season. In addition, broadleaved trees within or adjacent to villages were important activity areas for the breeding birds; protection and management measures should be increased in these areas.

## Introduction

A home range is an area that usually occurs around a home site and is traversed by an individual animal during its normal activities, such as food gathering, mating, and caring for the young. It provides an available space for the animals and allows it to achieve optimal fitness in the wild (Burt, 1943; Krebs & Davies, 1997; Kernohan, Gitzen & Millspaugh, 2001). Home range size is affected by factors such as animal sex, population density, predation risk and external environmental resources (Smith, 2007; Goltz et al., 2008; Hayes, Chesh & Ebensperger, 2011; Williams, Gutierrez & Whitmore, 2011). Resource availability determines home range size. Among such resources, space is determinant; for example, birds offset landscape constraints due to fragmentation by expanding their home range (see Godet et al., 2015). Habitat refers to a distinctive set of physical environmental factors that a species uses for its survival and reproduction, providing food, shelter, nesting locations and mating sites (Reunanen, Monkkonen & Nikula, 2002; Hall, Krausman & Morrison, 1997). Habitat use refers to the way in which an individual or species uses habitats to meet its daily needs (Block & Brennan, 1993; Krausman, 1999). The study of habitat use patterns involves the description of the actual distribution of individuals across habitat types (Hutto, 1985).

Studies on animal home range sizes, shape and utilization patterns can facilitate the understanding of interspecies relationships, population densities, habitat statuses and resource distribution (Mohr, 1947; Aogren, Zhou & Zhong, 1989; Mcintyre & Wiens, 1999; Dias & Strier, 2003). Such studies can also provide information for the evaluation of the quality and carrying capacity of the habitat, conservation area planning, ex situ conservation and reintroduction programmes (Marzluff et al., 1997; Woodroffe & Ginsberg, 2000; Garshelis, 2000; Stamps & Swaisgood, 2007).

The blue-crowned laughingthrush (*Garrulax courtoisi*,** hereafter BCLT), a critically endangered species in the family Leiotrichidae (BirdLife International, 2017), is 23–25 cm in length and weighs approximately 50 g; it is a monomorphic bird that lives in groups and feeds primarily on insects and other invertebrates. BCLTs migrate to breeding sites in middle or late April and breed colonially and cooperatively without territoriality. The nests are built in trees in/or around lowland villages; three or four eggs are laid in each clutch, incubation lasts approximately 12–13 days, and feeding of nestling lasts approximately 12–14 days (Liao et al., 2007). Sometimes reproduction lasts through late July due to a second brood, previous reproduction failure or first-time breeding (He et al., 2017). Both sexes build the nest, incubate eggs and brood nestlings during the whole breeding season (Wilkinson et al., 2004). The number of nests for the first clutch of a breeding flock would be no more than 1/3 of the number of birds in the flock (He et al., 2017). BCLTs leave the breeding site a few days after the young have fledged and roam throughout the nonbreeding season (Zhang et al., 2017).

Studies on this species have mostly focused on summaries of breeding habitat characteristics (Liao et al., 2007), probable movement range descriptions at one breeding site (Hong, Yu & Liao, 2006), and breeding habitat selection on a patch scale (Huang et al., 2018). However, most of these reports have been brief and incidental. The lack of knowledge on the ecology of this species could be detrimental to its conservation. Habitat protection is important for BCLTs*,* as some breeding sites are abandoned by birds, at least temporarily, when they are subjected to human-mediated disturbance (He et al., 2017), such as highway construction, tourism activity development, construction and housing renovations or new construction, river channel repair, etc.; disturbance from photographers has even caused BCLTs to abandon nest locations at their most important breeding sites (Zhang et al., 2017). However, as this species breed in an area also inhabited by humans, there are strong conflicts between habitat conservation and the demands of living environment transformation and production activities.

As far as we know, information regarding BCLTs’ home range size and utilization degree of different types of habitats within the breeding areas are completely lacking. It is crucial to provide such information, and thus, improve specific habitat management, such as protecting sufficiently large areas, identifying which areas to protect more stringently than others, relieving conflicts between BCLT and human activities, etc. Our study is the first providing these needed pieces of information about the very rare study species whose ecology and ethology are hardly known.

## Materials & Methods

### Study area

Wuyuan is located in northeastern Jiangxi Province, China. The coordinates are 29°01′–29°35′N, 117°22′–118°11′E, and the whole area encompasses 2947.51 km^2^. Wuyuan is located in a subtropical region where the climate is warm and humid, with an average annual temperature of 17.7 °C and average annual precipitation of 1330.3 mm. This area mainly consists of hills; the altitude varies from 50 to 1,600 m. It is rich in plant resources mainly composed of subtropical evergreen broadleaved trees, with a coverage rate as high as 82.6% (He et al., 2017). The Le’an River is the main river in Wuyuan, and its tributaries extend all over the country. Villages are situated along almost all the banks of the river in low-altitude areas, and the breeding sites of BCLTs occur in the Fengshui forests (mainly composed of broadleaved trees, such as Hackberry, *Celtis sinensis*; Chinese ash, *Pterocarya stenoptera*; and Chinese sweet gum *Liquidambar formosana*) in or beside the villages. Planted Fengshui forests are the main breeding areas of the BCLT in the villages. These patches are small in area and isolated from each other. 13 breeding sites were found in total from now on in Wuyuan (WW Zhang et al., 2019, unpublished data); 3 of these breeding sites that host large and stable breeding groups were chosen for home range study.

### Radio telemetry and home range calculations

**Figure 1:**
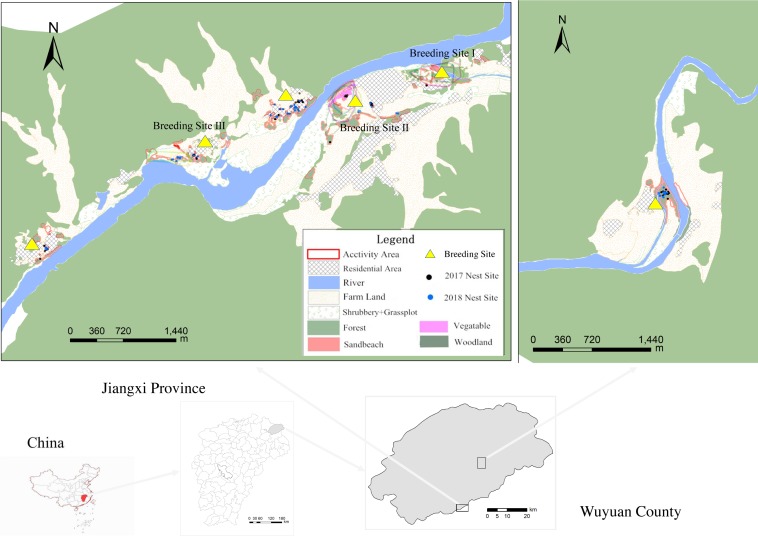
The relative positions and habitat features of the three breeding sites.

**Figure 2:**
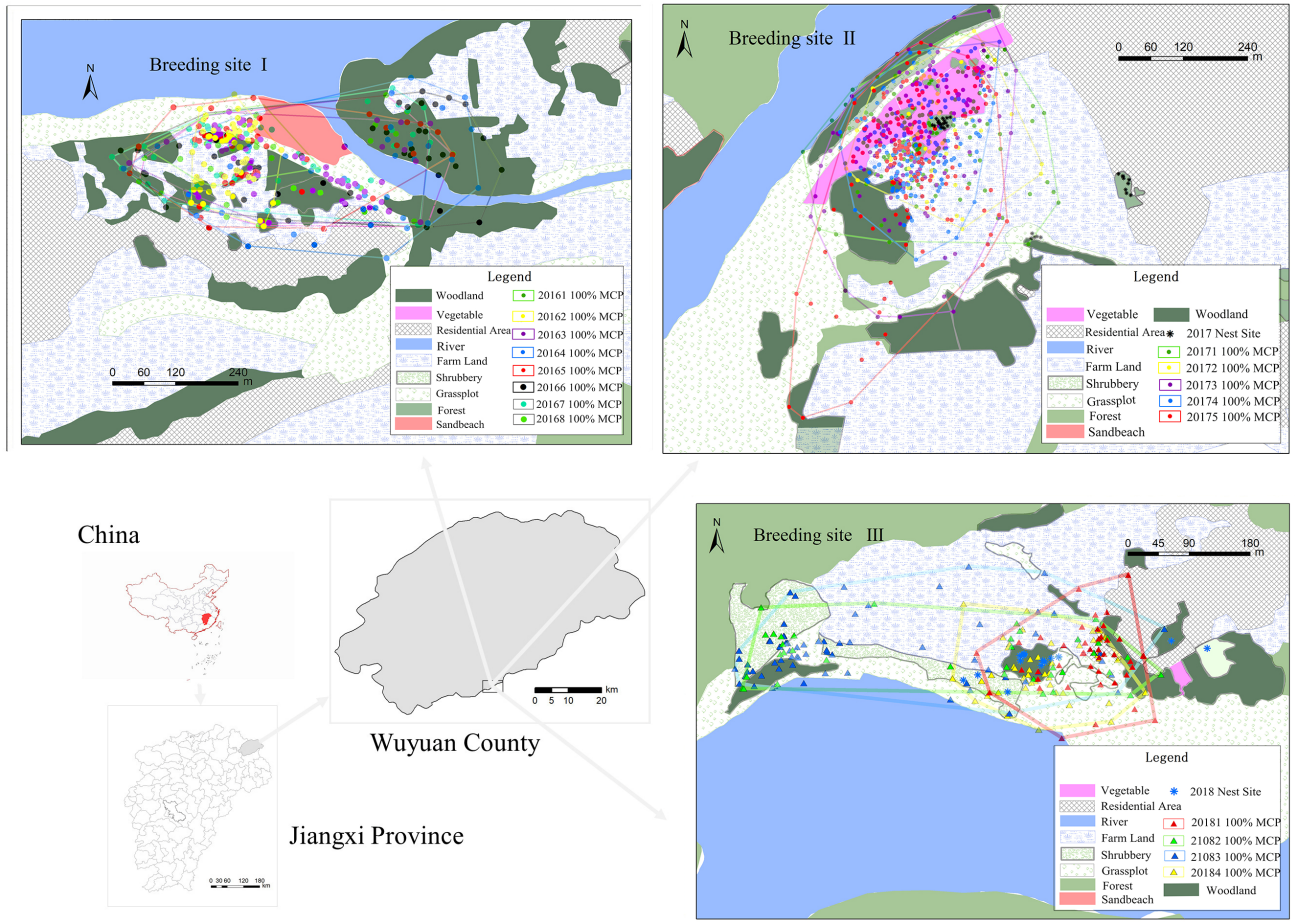
The radio telemetry study area and home range of the blue-crowned laughingthrush in 2016–2018.

The field experiments were approved by the Jiangxi Wildlife Conservation Administration (Jiangxi Forestry Office Copy No. 53 [2015]). Radio transmitters were fitted to the birds with the leg-loop harness method (Streby et al., 2015). The weight of the transmitters was approximately 1.2 g (A2455, ATStrack Corporation, USA), which was less than 3% of the body weight of the birds (55.99 g on average), and the battery life was approximately 30 days. We captured BCLTs at the breeding sites using mist nets in early May in 2016-2018 (breeding site I: 2016; breeding site II: 2017; breeding site III: 2018, see Figs. 1 and 2). The birds were marked with coloured aluminium metal leg rings and released. Signal reception started the next day with a hand-held radio receiver and folding antenna (LOTEK Corporation, USA). The positions of the birds were obtained according to the triangulation method or via direct observation (see Kolts & McRae, 2017). We tracked the birds from 7:00 to 11:00 h and from 15:00 to 18:00 h every day from early May to July (except on days with heavy rain or dense fog), and two telemetry positionings were performed every 30 min. The locations of each individual were documented at least 10 min apart to ensure independence. The intervals between collection during this period were determined based on the tracking schedules of concurrently tagged individuals and the distances between them (see the method in Kolts & McRae, 2017). We did not track the birds from 11:00–15:00 because of other fieldwork priorities and weather; during this time, the temperature was high, reaching above 40 °C, and the BCLTs were much less active than at other time. Even though we marked the tagged individuals, it was almost impossible to re-observe them in the field during the monitoring periods, as the nests were high in the trees, which had dense crowns, and the birds always moved in flocks. As a result, we could not confirm whether the labelled individuals reproduced or what was their reproductive status. As females were tagged, we inferred there were breeding individuals among them.

As the reception range of the equipment is less than 500 m, we attempted to navigate the nearest routes to approach the marked birds without disturbing them. We did this to reduce interference from the terrain or other factors with the signal and reduce variation in the active area due to human operation or equipment errors. Inaccurate positions (such as the middle of the river channel and high-altitude areas in hills) were excluded from the analysis according to our observations.

The home range area was represented by a 100% minimum convex polygon (MCP) (Hayne, 1949; Nilsen, Pedersen & Linnell, 2008). To facilitate future comparison between different studies, the 50–100% MCP, the 50–95% fixed KDE and core area of KDE were also obtained. The overlap degree between two individuals on home range or core area was measured with the overlap area size divide of the corresponded individual home range or core area.

### Habitat use and preference

The habitat use analysis were conduct both horizontally (i.e., landcover) and vertically (i.e., vertical stratification). We downloaded the Landsat-8 multi-band remote sensing image map of Wuyuan in the summer of 2017 from the USGS Global Visualization Viewer and combined it with a remote sensing image to obtain a true-colour image map of Wuyuan using ArcGIS 10.2. Habitat types were classified as water area, grass plot, shrubs, woodland, residential area, farmland, sand area and vegetable field. To investigate the preferred habitat types of BCLTs, we first extracted the composition of available habitats from the true-colour map for each individual by using 100% MCP home range contour using ArcGIS 10.2, and then the available area of each habitat was quantified. The frequency of the telemetry locations of each individuals in the different habitats were considered to represent the actual utilization of the different habitats.

We used a combination of line transect and point sampling method to investigate the vertical habitat selection of BCLTs. Fixed line transects (total length of 2-3 km) were established at the six breeding sites in 2018 and covered all habitat types. Observations for each fixed line transect were conducted twice a day from 7:00 to 11:00 h and from 15:00 to 18:00 h from early May to July using spotting scopes (Prostaff 7S, 10 × 42, Nikon Image Instrument Sales (China) Co., Ltd.), and the number of BCLTs and their vertical positions in the environment (horizontal and vertical scales) were recorded. The vertical position, including the ground (height 0–20 cm), shrub layer (20–100 cm), bottom of the trunk (100 cm-half trunk height), top of the trunk, and crown layer, were also recorded. For the point sampling method, we observed and recoreded the number for 15 min a period of time, and the instantaneous scanning method was used every 5 min to record the vertical position of the observed individual.

### Statistical methods

The 50–100% MCP and the fixed 50–95% KDE were obtained with the “adehabitatHR” package and Rhr package in R 3.6.1, href was used as smoothing parameter (Calenge, 2006; Signer & Balkenhol, 2015). The core area of KDE home range was determined by the method of (Seaman & Powell, 1990) by using the “rhr” package (Seaman & Powell, 1990; Signer & Balkenhol, 2015). The overlap degrees of the home range and core areas between different individuals were measured with the overlap index.

After testing for normality of the distribution, the differences in home range size (100% MCP and 95% KDE), estimated core area size, between the two sexes, as well as the available habitat area and actual utilization area were analysed by t-tests to evaluate the habitat selection in BCLTs (Hough & Dieter, 2009); Wilcoxon rank sums tests were used if the data were not normally distributed. ANOVA analysis was used to evaluate the differences among the three breeding sites.

For the vertical habitat utilization bias of the BCLTs, we compared the monthly mean frequency of each layer (use frequency/total use frequency).

All the data analyses were conducted in SPSS 17.0.****

## Results

### Home range characteristics of BCLT in the breeding season

Seventeen individuals were captured and banded from 2016–2018. The number, sex and tracking dates at each site of tracked individuals are shown in Table 1. A total of 1515 locations were obtained after 54 days of radio telemetry tracking (Table 1). The individual average 100% MCP for home range was 10.05 ± 1.17 ha, and the 95% KDE and KDE for the core area were 16.74 ± 2.05 and 7.84 ± 1.18 ha, respectively (Table 2). The other results regarding home range size are listed in supporting information files (File S3). The males’ average 100% MCP and 95% KDE for home range were 9.09 ± 1.232 ha and 19.72 ± 5.12 separately; these values was not significantly different from that of females (100% MCP = 11.62 ± 3.15 ha, *t* = 0.53, *df* = 14, *p* = 0.375; 95% KDE = 15.46 ± 2.35, *t* = 0.755, *p* = 0.489). There was also no significant difference in the estimated KDEs for the core areas between males (6.88 ± 1.19 ha) and females (7.97 ± 2.20 ha, *t* = 0.917, *df* = 14, *p* = 0.681) (Table 2). Moreover, there were no significant differences in the 100% MCPs and 95% KDE for the home ranges and core areas among three breeding sites (Table 2, Fig. 2).

**Table 1:** Radio telemetry, home range, and core area size data for the blue-crowned laughingthrush in 2016–2018.

ID	Sex	Breeding site	Capture date	Date of last transmission	Tracking period (days)	No. of locations	Home range size (100% MCP) (ha)	95% KDE Core area size	Estimated KDE Core area size
20161	♂	I	2016/5/11	2016/6/5	15	73	3.08	4.50	2.04
20162	♀	I	2016/5/12	2016/6/5	14	56	3.17	5.28	1.91
20163	♂	I	2016/5/14	2016/6/5	12	86	9.51	17.45	7.56
20164	?	I	2016/5/14	2016/6/5	12	43	15.42	20.06	18.87
20165	♀	I	2016/5/27	2016/6/5	10	90	11.06	22.06	7.53
20166	♀	I	2016/5/27	2016/6/5	10	80	14.22	29.45	11.33
20167	♂	I	2016/5/29	2016/6/5	8	72	11.23	23.39	11.65
20168	♂	I	2016/5/29	2016/6/5	8	71	11.48	20.06	9.65
20171	♂	II	2017/5/9	2017/6/10	22	176	13.70	14.41	6.80
20172	♂	II	2017/5/9	2017/6/10	22	72	8.08	11.16	6.33
20173	♂	II	2017/5/10	2017/6/10	21	156	16.74	18.00	8.29
20174	♂	II	2017/5/10	2017/6/10	21	181	8.20	9.34	5.42
20175	♀	II	2017/5/19	2017/6/20	17	157	18.02	22.07	11.09
20181	♂	III	2018/5/2	2018/6/10	17	43	4.11	6.59	3.22
20182	♂	III	2018/5/2	2018/6/10	17	37	6.59	30.41	2.87
20183	♂	III	2018/5/2	2018/6/10	17	73	12.58	23.94	15.86
20184	♂	III	2018/5/2	2018/6/10	17	49	3.72	6.34	2.81

**Table 2:** Average home range and core area size of the blue-crowned laughingthrush.

	*n*	100% MCP size (ha) (mean ± se)	*p* value	95% KDE core area size (ha) (mean ± se)	*p* value	Estimated KDE Core area size (ha)(mean ± se)	*p* value
I (2016)	8	9.90 ± 1.62	0.157	17.78 ± 3.07	0.858	8.82 ± 1.95	0.698
II (2017)	5	12.95 ± 2.08		14.99 ± 2.30		7.59 ± 0.99	
III (2018)	4	6.75 ± 2.05		16.82 ± 6.12		6.19 ± 3.23	
Males (2016–2018)	12	9.09 ± 1.23	0.375	19.72 ± 5.12	0.489	6.88 ± 1.19	0.681
Females (2016–2018)	4	11.62 ± 3.15		15.46 ± 2.35		7.97 ± 2.20	
All (2016–2018)	17	10.05 ± 1.17		16.74 ± 2.05		7.84 ± 1.18	

Forty-four overlaps for home range and core area were found among the 17 individuals; the average 100% home range overlap index was 62.86 ± 2.75%. Average overlap indexs for 95% KDE core area size and Estimated KDE core area size are also above 50% (File S3). Several pairs’ home range overlap were more than 80%, such as No.201605 (female), No.201607 (male) and No.201608 (male).

### Characteristics of habitat utilization

#### Habitat use determined from telemetry data

**Figure 3:**
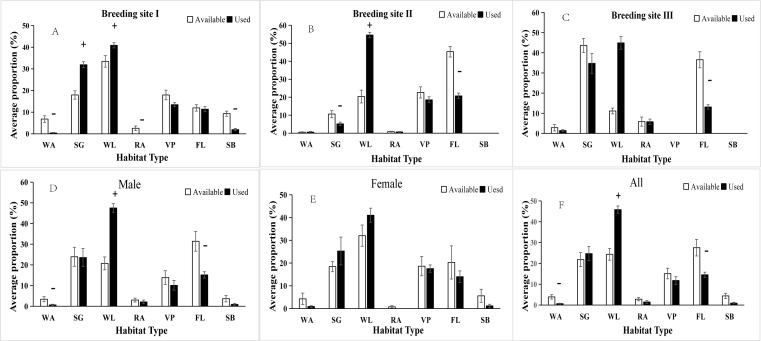
Average percentages of available and used habitat within breeding sites, different sexes and all blue-crowned laughingthrush individuals. (A) Breeding site I; (B) Breeding site II; (C) Breeding site III; (D) Male; (E) Female; (F) All. Habitat type WA: water area, SG: shrubs/grass plots, WL: woodland, RA: residential area, VP: vegetable plot, FL: Farmland, SB: sandy beach. A negative sign (“-”) indicates the ratio of used habitat was less than the available habitat, a plus sign (“+”) indicates that the ratio of used habitat was greater than the available habitat (according to independent-sample *t*-test, *P* < 0.05).

Overall, the BCLTs preferred woodland over the other types of habitats during the breeding season. The use ratio of woodland was 45.86 ± 1.74%; shrubs were the second most often used (24.72 ± 3.39%), followed by farmland (14.54 ± 1.24%), vegetable fields (11.80 ± 1.83%), residential areas (1.48 ± 0.67%), sand beach (0.92 ± 0.34%) and water areas (0.68 ± 0.22%). However, among all the available habitat types, the BCLTs showed a significant preference for woodland only (Fig. 3F).

The different sexes somewhat differed in terms of habitat use. Males preferred woodland mainly, and water areas and farmland were used very little (Fig. 3D); the females did not show any preference in terms of habitat utilization (Fig. 3E). There were some differences in habitat use across the three breeding sites, although woodland was always preferred. The birds at breeding site I were found more frequently in the shrubs and grass plots than in the other habitat types considering the availability.

#### Vertical habitat use based on visual observations

**Figure 4:**
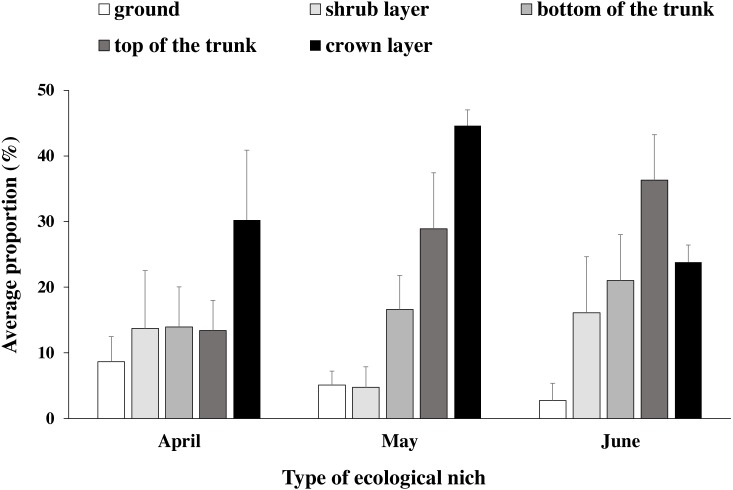
Blue-crowned Laughingthrush use of different ecological niches (mean percentage ± se).

Altogether, 6864 data points for habitat utilization were collected across 6 breeding sites from 17 April to 3 July. The arbor upper layer of the trees was used most frequently for nearly the whole breeding season, except in June, when the top of the trunk was most often used. The ground was used least overall; however, it was used more frequently in the early breeding season (April) than in the later breeding season, which was also the case for the shrub layer. The bottom of the trunk layer was increasingly used as the breeding season progressed (Fig. 4).

## Discussion

### Home range

Home range is empirically related to body weight (Harestad & Bunnel, 1979; Baker & Mewaldt, 1979; Jenkins, 1981), habitat composition and food availability (Santangeli et al., 2012; Kouba et al., 2017). The home range size of one group of Sumatran Laughingthrush (*G. bicolor*) with 5 individuals was 107 ha in North Sumatra, Indonesia; however, that value represented the home range size of this species in the nonbreeding season (Bušina & Kouba, 2017). The occupied territories of grey-crowned babblers (*Pomatostomus temporalis*) were approximately 2–53 ha (Blakers, Davies & Reilly, 1984) and increased with increasing group size (Counsilman, 1977). The yellow-billed babbler (*Argya affinis*) had a home range of 40 ha (Boby, 2001). The group home range size of the chestnut-crowned babbler (*P. ruficeps*) in the pre-nesting period was 121.7 ha on average, during nesting it decreased to 56.6 ha (Sorato, Griffith & Russell, 2016).

All individuals of the three breeding sites each year showed overlap with each other (Fig. 2, File S3), indicating the BCLTs still lived in groups in breeding season, not breeding pair or family group. As the babblers always moved in a group, their home ranges were always measured as groups and differed according to group sizes, seasons, food source impacts and habitat structures. However, even though the home range of the BCLT seems smaller than those of other kin species, the BCLT roams in the nonbreeding season; therefore, we suspect that their activity range is much larger than the value we calculated during the breeding season.

The home ranges of female white-crowned sparrows (*Zonotrichia leucophrys*) are smaller than those of males in summer but larger in winter. The females build nests, incubate eggs and brood the nestlings alone during the breeding period; this has been considered to reduce the females’ activity frequency and scope (Baker & Mewaldt, 1979). However, there were no significant differences in the home ranges of the different sexes of *G. courtoisi*, as they build nests, incubate eggs and brood nestlings together during the whole breeding season; as members of a cooperative breeding species (Wilkinson et al., 2004), they flock and move together during the breeding season. The parents alternately forage and feed the chicks and sometimes the helpers; as a result, individuals of different statuses and sexes have high overlap in terms of their home range. Furthermore, their weak long-distance migration ability and abundant food availability (WW Zhang, 2019, unpublished data) indicate that only a small area is necessary to meet their needs for survival and reproduction. Therefore, we can infer that suitable habitat area is not a limiting factor in terms of population increase in the BCLT.

Among the three breeding sites, the average individual home range at site II was significantly larger than that at site III. The size of the home range is strongly linked to habitat quality and food resource richness (Smith, 2007). The main habitat and feeding areas of laughingthrushes are woodland and shrubs (Zhao, 2001; Huang et al., 2018). Energy costs and predation risks increase as the feeding distance increases (Brown, 1988; Tsurim et al., 2010). Therefore, breeding in habitats rich in food resources and avoiding long-distance movement may be survival strategies of the BCLT. The available woodland and shrub area at breeding site III (54.76%) was significantly larger than that at site II (30.99%). Therefore, we inferred that the food resources at breeding site III were better than those at site II, leading to the shorter movement distance and smaller home range.

### Habitat utilization

#### Horizontal scale

How breeding birds are distributed in relation to landscape-scale habitat features has important implications for conservation because those features may constrain habitat suitability (Anteau et al., 2014). Woodland, with a good nesting environment, abundant food resources and concealing conditions, is an important habitat type for many birds (Nicholls & Warner, 1972; Anders, Faaborg & Al, 1998; Streby, Loegering & Andersen, 2012) and an important stopover area during migration (Mehlman et al., 2005; Rush, Soehren & Miller, 2014). The BCLTs greatly rely on the Fengshui forests within or near the villages (Liao et al., 2007). The dominant species in the Fengshui forests are broadleaved trees, which also represent the main nesting area for the BCLT and a food resource during the breeding season. They build their nests in high trees with a large diameter at breast height or small trees with dense branches (i.e., Sweet Osmanthus *Osmanthus fragrans*); they also sometimes nest in bamboo, densely planted seedling forests, or fruit trees in residential areas but never in shrubs or brush (Hong, Yu & Liao, 2006; He et al., 2017; Huang et al., 2018).

The feeding habitat of the BCLT is diverse; they feed in the crown layer and trunks of trees, shrubs under the trees or at the forest edge, grassland, tea gardens, vegetable fields, shrubs along riverbanks, and scattered trees at the breeding sites. They feed in almost all kinds of habitats except open areas and farmland (Hong, Yu & Liao, 2006; this study). Shrubs are also used as “steppingstones” when the birds are flying from one patch to another near the feeding areas. We observed that the BCLTs quickly flew into the shrubs when raptors appeared. The use of farmland mainly occurred during the initial stage of the breeding season. The birds searched the soil for soil organisms in the fields, which were always rape fields. We also observed the BCLTs searching for nest-building materials in the farmland. The sand and water areas were used mainly for washing and cleaning.

The males, but not the females, showed a significant preference for woodland and little utilization of water areas or farmland that were large in size in or near the breeding sites, indicating that woodland was the most preferred habitat, providing more food and better concealment than the other habitat types. The observed difference between the sexes might have been caused by the biased sample, as males made up a higher proportion of our marked individuals than females.

#### Vertical scale

The BCLTs utilized the crown layer of the trees most often during the breeding season, while the ground was used most often in the early period of the breeding season. In mid-April, the BCLTs arrived at the breeding sites, and we observed that they searched for food in the grass or in the soil; consequently, their beaks were always covered in clay. The shrubs were used as steppingstones when the birds flew from one place to another or from the ground to the trees. Therefore, the utilization ratio of the shrubs and the tea bushes was highest in April. When the birds began to build their nests, more activity was observed among the different tree layers, especially the crown layer, where the nests were located, and the upper layer of the trees was used less after the nestlings fledged. Most timaliids, such as the masked laughingthrush (*G. perspicillatus*) (Li et al., 2017), giant laughingthrush (*G. maximus*) (Wang et al., 2010), Elliot’s laughingthrush (*G. elliotii*) (Opaev, Liu & Kang, 2017), and white-crested laughingthrush (*G. leucolophus*)** (Collar & Robson, 2007) nest in brush, shrubs, dwarf trees or bamboo; the nest height above the ground is approximately or less than 4–5 m, which is lower than that for *G. courtoisi*, whose nest height is approximately 14 m (Zhang et al., 2017). In contrast to other timaliids, *G. courtoisi* is highly dependent on tall broadleaf trees, especially their dense canopies, which forms a special microhabitat with good shelter and foraging conditions*.*

The BCLTs’ activity areas were fairly limited in the breeding season, but the habitat composition was very complex, and human-mediated elements, such as vegetable gardens, farmland, bamboo stands, and tea gardens are important to breeding groups. Compared with the preservation of large areas of continuous forest, the preservation of small areas of woodland with multiple types of vegetation is essential for the conservation of the BCLT. However, as the country development, human disturbance and environment transformation have negatively affected its habitat (He et al., 2017; Zhang et al., 2017). As a result, we need to control the disturbance to or destruction of these areas to protect this species effectively.

## Conclusions

The home range of the BCLT was limited to small areas during the breeding period. The overlap degree was high between different individuals in the same sites, as they still moved in a group during the reproductive period. We strongly recommend protection and management of the trees within or beside the villages utilized by *G. courtoisi*, as well as bamboo stands, shrubs and vegetable plots.

##  Supplemental Information

10.7717/peerj.8785/supp-1 File S1 2016: The original and processed data of radio telemetry

10.7717/peerj.8785/supp-2 File S2 2018: The original and processed data of radio telemetry

10.7717/peerj.8785/supp-3 File S3 Home range size of the BCLT and overlap results

10.7717/peerj.8785/supp-4 File S4 2017: The original and processed data of radio telemetry

10.7717/peerj.8785/supp-5 File S5 Available and used habitat of the BCLT

10.7717/peerj.8785/supp-6 File S6 The original data of ecological niche using frequency by BCLT

## Additional Information and Declarations

### Field Study Permissions

The following information was supplied relating to field study approvals (i.e., approving body and any reference numbers): Field experiments were approved by the Jiangxi Wildlife Conservation Administration (Jiangxi Forestry Office Copy No. 53 [2015]).

### Data Availability

The following information was supplied regarding data availability: The raw measurements are available in the Supplemental Files.
